# Nonmotor symptom burden grading as predictor of cognitive impairment in Parkinson’s disease

**DOI:** 10.1002/brb3.2086

**Published:** 2021-03-01

**Authors:** Panteleimon Oikonomou, Daniel J. van Wamelen, Daniel Weintraub, Dag Aarsland, Dominic Ffytche, Pablo Martinez‐Martin, Carmen Rodriguez‐Blazquez, Valentina Leta, Corinne Borley, Carolina Sportelli, Dhaval Trivedi, Aleksandra M. Podlewska, Katarina Rukavina, Alexandra Rizos, Claudia Lazcano‐Ocampo, Kallol Ray Chaudhuri

**Affiliations:** ^1^ Department of Neurosciences King’s College London Institute of Psychiatry, Psychology & Neuroscience London UK; ^2^ Parkinson Foundation Centre of Excellence at King's College Hospital London UK; ^3^ Department of Neurology and Neurophysiology Medical Center‐University of Freiburg Freiburg Germany; ^4^ Department of Neurology Donders Institute for Brain, Cognition and Behaviour Radboud University Medical Centre Nijmegen the Netherlands; ^5^ Perelman School of Medicine University of Pennsylvania Philadelphia PA USA; ^6^ Parkinson's Disease Research Education and Clinical Center (PADRECC) Philadelphia Veterans Affairs Medical Center Philadelphia PA USA; ^7^ Department of Old Age Psychiatry Institute of Psychiatry, Psychology & Neuroscience King's College London London United Kingdom; ^8^ Center for Networked Biomedical Research in Neurodegenerative Diseases (CIBERNED) Carlos III Institute of Health Madrid Spain; ^9^ National Centre of Epidemiology and CIBERNED Carlos III Institute of Health Madrid Spain; ^10^ Department of Neurology Hospital Sotero del Río Santiago de Chile Chile

**Keywords:** cognitive impairment, MMSE, Nonmotor symptom burden grading, Nonmotor symptoms, Parkinson's disease

## Abstract

**Background:**

Identifying predictors of incident cognitive impairment (CI), one of the most problematic long‐term outcomes, in Parkinson's disease (PD) is highly relevant for personalized medicine and prognostic counseling. The Nonmotor Symptoms Scale (NMSS) provides a global clinical assessment of a range of NMS, reflecting NMS burden (NMSB), and thus may assist in the identification of an “at‐risk” CI group based on overall NMSB cutoff scores.

**Methods:**

To investigate whether specific patterns of PD NMS profiles predict incident CI, we performed a retrospective longitudinal study on a convenience sample of 541 nondemented PD patients taking part in the Nonmotor Longitudinal International Study (NILS) cohort, with Mini‐Mental State Examination (MMSE), NMSS, and Scales for Outcomes in PD Motor Scale (SCOPA Motor) scores at baseline and last follow‐up (mean 3.2 years) being available.

**Results:**

PD patients with incident CI (i.e., MMSE score ≤ 25) at last follow‐up (*n* = 107) had severe overall NMSB level, significantly worse NMSS hallucinations/perceptual problems and higher NMSS attention/memory scores at baseline. Patients with CI also were older and with more advanced disease, but with no differences in disease duration, dopamine replacement therapy, sex, and comorbid depression, anxiety, and sleep disorders.

**Conclusions:**

Our findings suggest that a comprehensive baseline measure of NMS and in particular hallucinations and perceptual problems assessed with a validated single instrument can be used to predict incident CI in PD. This approach provides a simple, holistic strategy to predict future CI in this population.

## Graphical abstract

A comprehensive baseline measure of NMS and in particular hallucinations and perceptual problems assessed with a validated single instrument can be used to predict incident cognitive impairment in PD. This approach provides a simple, holistic strategy to predict future cognitive impairment in this population.

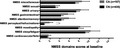

## 1 INTRODUCTION

Cognitive impairment (CI) is one of the most prominent and clinically relevant nonmotor features in Parkinson's disease (PD) (Aarsland et al., 2017), being an indicator for poor quality of life for patient as well as carers and having a significant impact on societal and institutionalization related costs (Goldman et al., 2018). The spectrum ranges from subtle cognitive changes, through mild CI (PD‐MCI) with no significant difficulties of daily living, to PD dementia (PDD) with substantially affected daily functioning and a greater degree and variety of cognitive deficits (Aarsland et al., 2017). The identification of predictors of CI is highly relevant for (a) personalized management strategies (e.g., advanced counseling, avoiding anticholinergics, and earlier use of cholinesterase inhibitors) (Titova & Chaudhuri, 2017) and (b) enriching trial populations for potential neuroprotection and palliative care (Martinez‐Martin & Ray Chaudhuri, 2018). Based on the available evidence, several clinical and demographic factors such as higher age at PD onset, fewer years of formal education, increasing severity of disease, and psychiatric disorders (e.g., depression and psychosis) predict future development of PDD (Anang et al., 2014; Liu et al., 2017; Marinus et al., 2018; Szatmari et al., 2017).

An approach to address the development of potential CI in PD, using for example a validated and widely used NMS burden (MNSB) grading system (Goldman et al., 2018; Ray Chaudhuri et al., 2013) seems intuitively reasonable, given the reported links of CI with disease severity (Anang et al., 2014; Goldman et al., 2018; Liu et al., 2017) clinical subtypes (Marras & Chaudhuri, 2016), neuropathological burden (Halliday et al., 2014), and drug treatment (NMSB grading may also reflect drug‐induced NMS for instance) (Goldman & Weintraub, 2015). NMSB grading provides a simple, yet comprehensive method for quantifying PD NMS load (Martinez‐Martin, 2013) and can be used as a clinical biomarker (Martinez‐Martin & Ray Chaudhuri, 2018). The PD Nonmotor Symptoms Scale (NMSS) remains the only scale (recently updated as MDS‐NMS) as a specific measure of a range and nature of NMS and validated cutoffs for NMSB have been published (Chaudhuri et al., 2007; Ray Chaudhuri et al., 2013).

In an effort to identify possible clinical predictors of CI in PD using one comprehensive tool, we aimed to explore two issues: (a) which out of the nine NMSS domains are associated with CI in PD patients, using a large‐scale cohort and a “real‐life” data mining‐based analysis and (b) does a higher NMSB at baseline predict to CI after 3 years. Our hypothesis was that the burden of specific NMS and total NMSB in a large cohort of PD patients could be different in those who developed CI at follow‐up from those who did not.

## 2 METHODS

For this analysis, we selected a longitudinal dataset of 541 consecutive PD patients taking part in the Nonmotor Longitudinal International Study (NILS) at King's College Hospital for whom Mini‐Mental State Examination (MMSE) scores were available and who had at least one follow‐up assessment as part of NILS. NILS was adopted by the National Institute of Health Research in the United Kingdom (UKCRN No. 10084) as the first comprehensive longitudinal study identifying nonmotor profiles in PD, as well as the natural history of NMS, treatment response, and clinic‐pathological‐imaging correlations. The study was authorized by local ethics committees (NRES SouthEast London REC3, 10084, 10/H0808/141). All patients gave written consent prior to study procedures in accordance with the Declaration of Helsinki and Good Clinical Practice.

Data were analyzed from a cumulative cohort of PD patients recruited between November 2011 (start of NILS data collection) and July 2019 (data extracted on 1 July 2019), and only data from patients included in the United Kingdom were analyzed. The main inclusion criterion was diagnosis of idiopathic PD according to the UK Brain Bank criteria. We only included data from the baseline assessments and at last follow‐up in the analysis. All included patients were nondemented at baseline as defined by an MMSE score ≥28 (O'Bryant et al., 2008). Exclusion criteria were (1) diagnosis of Parkinsonism different to idiopathic PD and (2) inability to give consent to participate in the study. The patient cohort was divided into two groups based on the MMSE scores at follow‐up: cognitively normal (CN) (MMSE score of ≥26) or cognitively abnormal (CA) (MMSE score of ≤ 25) (Dubois et al., 2007).

Demographic data of the included PD patients contained information regarding age, sex, disease duration, and duration of follow‐up. In our analysis, we used data from Hoehn and Yahr (HY) staging (Hoehn & Yahr, 1967), NonMotor Symptoms Scale (NMSS), levodopa equivalent dose (LEDD) (Tomlinson et al., 2010), MMSE (Folstein et al., 1975), and SCales for Outcomes in PArkinson's disease (SCOPA)‐MOTOR (Marinus et al., 2004), comprising of ‐motor examination (SCOPA‐ME, activities of daily living (SCOPA‐ADL), and motor complications (SCOPA‐MCompl) assessments. The NMSS facilitates a rater‐administered comprehensive assessment of NMS in PD patients and includes 30 items grouped in nine relevant domains: (1) cardiovascular including falls, (2) sleep/fatigue, (3) mood/apathy, (4) perceptual problems/hallucinations, (5) attention/memory, (6) gastrointestinal tract, (7) urinary function, (8) sexual function, and (9) miscellaneous. The NMSS Score for each item is based on a multiplication of severity (from 0 to 3) and frequency (from 1 to 4) scores (Martinez‐Martin & Ray Chaudhuri, 2018). Furthermore, we included data from patient‐reported outcomes (i.e., Hospital Anxiety and Depression Scale (HADS‐total); a 14‐item, patient‐completed scale with subscales for anxiety and depression (Zigmond & Snaith, 1983); PD Sleep Scale‐version 1 (PDSS) and a 15‐item, patient‐completed clinical tool used to assess the frequency of sleep disturbances during the past week in PD patients) (Chaudhuri et al., 2002). Using overall NMSS scores, the levels of NMSB were determined based on the validated cutoffs of the published NMSB grading system (Ray Chaudhuri et al., 2013). NMSS total score of 0 is related to “no,” 1–20 to “mild,” 21–40 to “moderate,” 41–70 to “severe,” and ≥71 to “very severe” NMSB level (Ray Chaudhuri et al., 2013).

Data are represented as mean and standard deviation, median [interquartile range], or number (percentage), unless otherwise specified. Group differences were tested using the Mann–Whitney test, and intragroup differences (baseline to follow‐up) were tested using the Wilcoxon signed rank test, as the data used in this study were not normally distributed (*p* ≤.001; Shapiro–Wilk test). The significance threshold was set at 0.05. A Quade's rank analysis of covariance was performed to correct for statistically significant differences in age between the two groups at baseline, and a Benjamini‐Hochberg correction was used in case of multiple comparisons. To test for differences of gender and NMSB levels, Pearson's chi‐square analysis was used. To estimate the association between the score of baseline clinical evaluations and the incident CI at follow‐up, two binary logistic regression models were performed, using the dichotomized MMSE at follow‐up defined as normal (≥26) and abnormal (<26) as dependent variable. The independent variables in the first model were LEDD, PD duration, PDSS, SCOPA Motor, and NMSS total scores at baseline. In the second model, the NMSS domains scores at baseline replaced NMSS total scores. The rest of variables were not included due to possible collinearity. Both regression models were adjusted for age and gender. All data were analyzed using SPSS Version 25 (IBM SPSS Statistics for Windows, Version 25.0. Armonk, NY: IBM Corp).

## 3 RESULTS

Of the 541 patients of our study, 434 had normal cognitive function at follow‐up (CN group) and 107 had CI (CA group). Mean duration of follow‐up was 3.18 ± 1.48 years (minimum 0.6, maximum 6.9 years) for the CN and 3.28 ± 1.79 years (minimum 0.4 years, maximum 7.2 years) for the CA group. At baseline, the 434 patients in the CN group had mean age 64.44 ± 11.27 years, disease duration 5.43 ± 5.21 years, median HY stage 2 [1.0–3.0], and NMSS total score 45.19 ± 35.29. 24.1% (*n* = 104) of these patients had mild, 30.8% (*n* = 133) moderate, 24.1% (*n* = 104) severe, and 19.9% (*n* = 86) very severe NMSB level at baseline. Patients in CA group (*n* = 107) had a mean age of 70.66 ± 8.64, disease duration of 5.63 ± 5.36) years, median HY stage 2 [2.0–3.0], and NMSS total score 52.76 ± 40.97 at baseline. 24.3% (*n* = 26) of these patients had mild, 24.3% (*n* = 26) moderate, 19.6% (*n* = 21) severe, and 30.8% (*n* = 33) very severe NMSB level at baseline. The two groups were well matched regarding gender (*p* = .15), duration of disease (*p* = .79), follow‐up (*p* = .74), and LEDD (*p* = .66). Furthermore, as per inclusion criteria, all patients were nondemented at baseline, as defined by MMSE of ≥28. Importantly, no statistical differences were found in total NMSS scores between groups at baseline (*p* = .41), nor in distribution of NMSB grading (*p* = .15). Nonetheless, patients from the CA group were significantly older (*p* <.001) and showed, moreover, significantly higher scores in SCOPA‐ME (*p* = .004), SCOPA‐ADL (*p* = .004) compared with the CN patients at baseline. (Table 1).

**TABLE 1:** Descriptive statistics of the study groups at baseline and at follow‐up

	Baseline				Follow‐up			
	CA (*n* = 107)	CN (*n* = 435)	*p* *	*p* ^†^	CA (*n* = 107)	CN (*n* = 435)	*p* *	*p* ^†^
Baseline demographics								
Age (ys)	70.66 ± 8.64	64.44 ± 11.27	**<.001**	N/A	73.78 ± 8.46	67.72 ± 11.23	**<.001**	N/A
Gender (M/F)	69.2%/30.8%	61.8%/38.2%	.159	.318	69.2%/30.8%	61.8%/38.2%	.159	.237
Disease duration (ys)	5.63 ± 5.36	5.43 ± 5.21	.798	.798	8.81 ± 5.85	8.72 ± 5.36	.856	.731
Duration follow‐up (ys)	3.18 ± 1.48	3.28 ± 1.79	.743	.798	N/A	N/A	N/A	N/A
LEDD (mg)	473.37 ± 407.35	512.70 ± 475.03	.667	.798	728.02 ± 460.96	694.20 ± 468.33	.452	.237
HY^‡^	2.0 [2.0–3.0]	2.0 [1.0–3.0]	**.023**	.061	3.0 [2.0–3.0]	2.5 [2.0–3.0]	**<.001**	**.019**
Outcome measures								
SCOPA‐ME	11.36 ± 5.36	9.40 ± 4.89	**.001**	**.004**	13.65 ± 5.55	10.35 ± 5.11	**<.001**	**<.001**
SCOPA‐ADL	6.23 ± 3.45	4.98 ± 3.29	**.001**	**.004**	8.93 ± 3.80	6.46 ± 3.82	**<.001**	**<.001**
SCOPA‐MCompl	1.68 ± 2.82	1.61 ± 2.52	.592	.798	1.98 ± 2.12	2.37 ± 2.42	.196	.639
NMSS cardiovascular/falls	1.63 ± 2.91	1.45 ± 2.47	.642	.963	2.27 ± 3.60	1.61 ± 2.68	**.016**	**.033**
NMSS sleep/fatigue	10.20 ± 10.32	9.16 ± 8.45	.771	.973	11.17 ± 9.08	9.40 ± 9.02	**.035**	**.033**
NMSS mood/apathy	8.10 ± 11.21	7.74 ± 11.63	.892	.973	10.12 ± 12.43	7.23 ± 11.85	**.004**	**.010**
NMSS perceptual/hallucinations	1.84 ± 3.82	0.76 ± 2.05	**.002**	**.024**	3.44 ± 5.02	1.77 ± 3.72	**<.001**	**.003**
NMSS attention/memory	5.79 ± 6.90	4.27 ± 5.80	**.034**	.204	8.81 ± 8.45	4.79 ± 6.79	**<.001**	**<.001**
NMSS gastrointestinal	5.58 ± 6.71	4.20 ± 5.35	.071	.284	6.06 ± 6.23	4.77 ± 5.70	**.038**	.228
NMSS urinary	8.20 ± 8.77	7.29 ± 8.00	.322	.552	8.56 ± 9.29	7.88 ± 8.80	.435	.793
NMSS sexual	3.01 ± 5.06	3.09 ± 5.66	.892	.973	1.88 ± 4.70	1.79 ± 4.38	.781	.797
NMSS miscellaneous	8.43 ± 8.57	7.11 ± 7.42	.223	.454	8.60 ± 8.50	7.03 ± 6.72	.180	.103
NMSS total	52.76 ± 40.97	45.19 ± 35.29	.139	.417	60.72 ± 43.11	46.26 ± 37.90	**<.001**	**.003**
PDSS total	109.92 ± 27.48	107.36 ± 25.51	.227	.454	95.23 ± 29.07	100.41 ± 25.82	.135	.228
HADS total	11.01 ± 7.44	10.77 ± 6.47	.985	.985	13.97 ± 7.69	11.29 ± 6.81	**.001**	**.003**

Note

Data are represented as mean ± standard deviation, unless otherwise specified. Group differences tested using Mann–Whitney *U* test.

ADL, activities of daily living; CA, cognitively abnormal (MMSE score of ≤25 at follow‐up); CN, Cognitively normal (MMSE score of ≥26 at follow‐up); F, female; HADS, hospital anxiety and depression scale; HY, Hoehn and Yahr; LED, Levodopa equivalent dose; M, male; MCompl, motor complications; ME, motor examination; *N*, number; NMSS, nonmotor symptom scale; PDSS, Parkinson's disease sleep scale; SCOPA, SCales for Outcomes in PArkinson's disease; Ys, years.

* Uncorrected *p*‐values.

^†^ *p*‐values corrected for age (Quade's rank analysis of covariance correction) and multiple testing (Benjamini‐Hochberg procedure).

^‡^ MEDIAN [25th–75th percentile].

**FIGURE 1:**
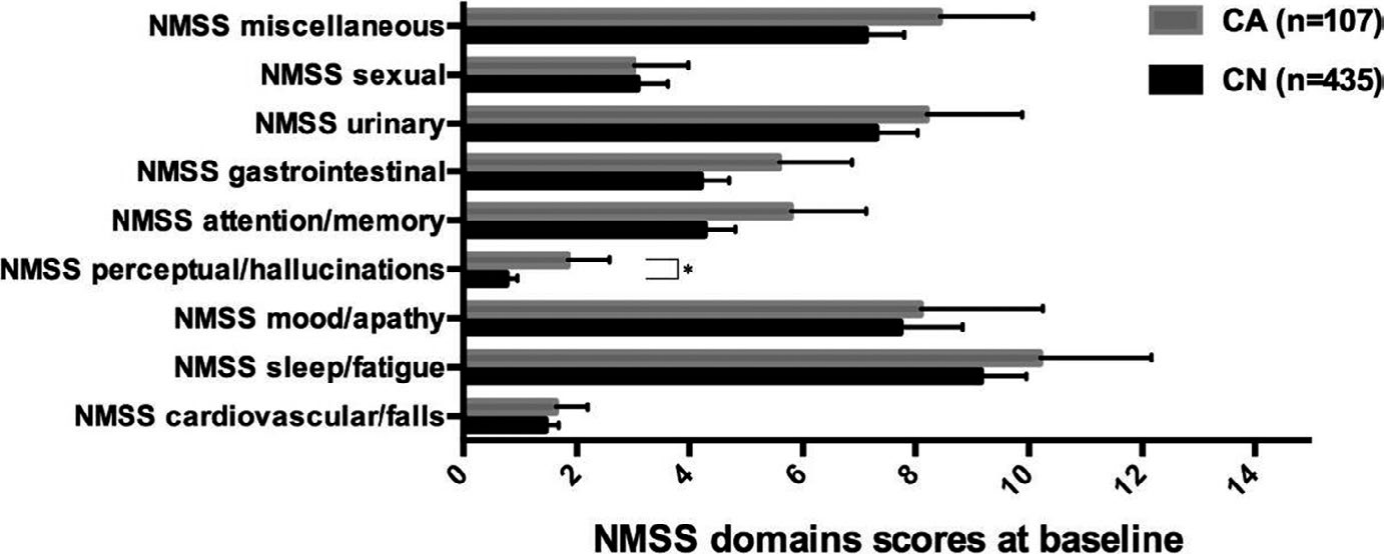
NMSS domains scores between the study groups at baseline. Data presented as mean and 95% confidence intervals (bars). CA, cognitively abnormal (MMSE score of ≤ 25 at follow‐up); CN, Cognitively normal (MMSE score of ≥26 at follow‐up). * Indicates a *p* value of .024 (Quade's rank analysis of covariance correction for age and Benjamini‐Hochberg procedure correction for multiple testing); The NMSS Score for each item is based on a multiple of severity (from 0 to 3) and frequency (from 1 to 4) scores

In terms of the NMSS domain scores at baseline, the patients of the CA group had significantly higher scores in domain 4 (perceptual problems/hallucinations) (*p* = .024) compared with the CN patients. No significant differences between the two groups of patients were found at baseline in domains 1 (cardiovascular), 2 (sleep/fatigue), 3 (mood/apathy), 6 (gastrointestinal tract), 7 (urinary function), 8 (sexual function), and 9 (miscellaneous) of NMSS (*p* ≥.48) or in HADS‐total, SCOPA‐MCompl, and PDSS scores (*p* ≥.45). No significant differences were also found in NMSS domain 5 (attention/memory) (*p* = .2) and HY stage (*p* = .06) despite the trend toward statistical significance founded in the analysis without correction for age and multiple testing. (Figure 1).

**FIGURE 2:**
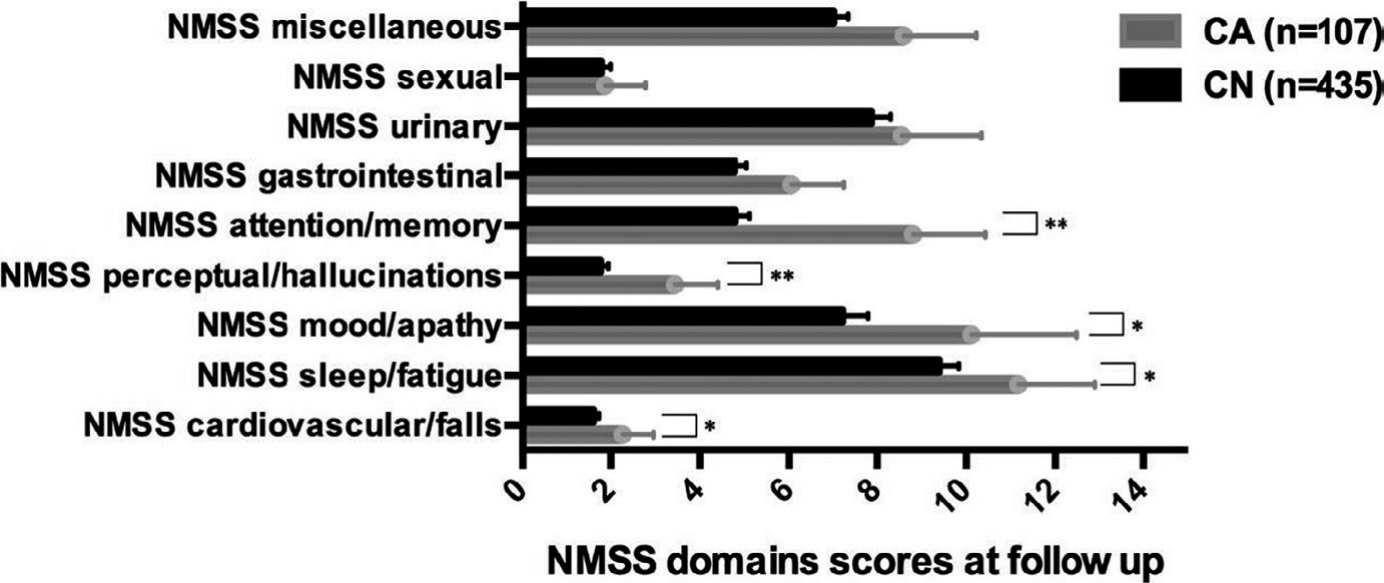
NMSS domains scores between the study groups at follow‐up. Data presented as mean and 95% confidence intervals (bars). CA, cognitively abnormal (MMSE score of ≤ 25 at follow‐up); CN, Cognitively normal (MMSE score of ≥ 26 at follow‐up). * Indicates a *p* value <.05 (Quade's rank analysis of covariance correction for age and Benjamini‐Hochberg procedure correction for multiple testing); ** indicates a *p* value <.005 (Quade's rank analysis of covariance correction for age and Benjamini‐Hochberg procedure correction for multiple testing); The NMSS Score for each item is based on a multiple of severity (from 0 to 3) and frequency (from 1 to 4) scores

At follow‐up, patients in the CA group showed significantly higher median HY scores (3.0 [2.0–3.0] vs. 2.5 [2.0–3.0]: *p* = .019), NMSS total scores (60.72 ± 43.11 vs. 46.26 ± 37.90: *p* = .003), NMS cardiovascular domain scores (2.27 ± 3.60 vs. 1.61 ± 2.68: *p* = .033), sleep/fatigue domain scores (11.17 ± 9.08 vs. 9.40 ± 9.02: *p* = .033), mood/apathy domain scores (10.12 ± 12.43 vs. 7.23 ± 11.85: *p* = .010), perceptual problems/hallucinations domain scores (3.44 ± 5.02 vs. 1.77 ± 3.72: *p* = .003), attention/memory domain 5 scores (8.81 ± 8.45 vs. 4.79 ± 6.79: *p* <.001), as well as of SCOPA‐ME scores (13.65 ± 5.55 vs. 10.35 ± 5.11: *p* <.001), SCOPA‐ADL scores (8.93 ± 3.80, vs. 6.46 ± 3.82: *p* <.001), and HADS‐total scores (13.97 ± 7.69 vs. 11.29 ± 6.81: *p* = .003) compared to CN patients. No significant differences were observed in any of the other used clinical assessments. (Figure 2).

In order to identify the important baseline predictive factors of relevant CI at follow‐up, we designed two binary regression models. In the first regression model, retained variables were age (odds ratio, OR: 1.06; 95% confidential interval, 95% CI: 1.03–1.08) and SCOPA‐ME (OR: 1.07; 95% CI: 1.02–1.11) at baseline. In the second model, NMSS domain 4 (perceptual problems/hallucinations) scores at baseline were retained (OR: 1.10; 95% CI: 1.02–1.19) together with age (OR: 1.05; 95% CI: 1.03–1.08) and SCOPA‐ME (OR: 1.06; 95% CI: 1.01–1.10) at baseline. (Table 2).

**TABLE 2:** Results of logistic regression models

	B	*p*‐value	OR	95% C.I. for OR	
				Lower	Upper
First Model					
Age	0.055	<.001	1.057	1.033	1.081
SCOPA‐ME	0.067	.002	1.069	1.025	1.116
Constant	−5.828	<.001	0.003		
Second Model					
Age	0.053	<.001	1.055	1.031	1.079
SCOPA‐ME	0.054	.017	1.056	1.010	1.103
NMSS domain 4	0.097	.014	1.102	1.020	1.191
Constant	−5.681	<.001	0.003		

Note

First model: Dependent variable; the dichotomized MMSE at follow‐up defined as MMSE_FU_REC: 0 = normal (≥26); 1 = abnormal (<26). Independent variables were Levodopa equivalent dose (LED), PD duration, Parkinson's disease sleep scale (PDSS), SCales for Outcomes in PArkinson's disease ‐motor examination (SCOPA‐ME) and nonmotor symptom scale (NMSS) total scores at baseline. Second model: Dependent variable; defined as MMSE_FU_REC: 0 = normal (≥26); 1 = abnormal (<26). Independent variables LED, PD duration, PDSS, SCOPA‐ME and NMSS domains scores at baseline. Only data for significant predictors are shown.

B, Beta value; C.I., confidence interval; NMSS domain 4, perceptual problems/hallucinations; OR, Odds Ratio.

## 4 DISCUSSION

In this large‐scale, longitudinal cohort‐based retrospective analysis, we showed that:

1. PD patients who developed CI over the 3.2 years follow‐up period had significantly worse NMSS baseline scores for hallucinations/perceptual problems with no baseline intergroup differences in disease duration, dopaminergic medication, gender and presence of depression, anxiety, and sleep disorders.
2. Higher burden of hallucinations/perceptual problems, but not overall nonmotor burden at baseline, predicted CI in PD, which suggests that these symptoms are likely to precede CI, as measured by objective screening tools such as the MMSE.

We believe that this may be the first study which examined whether CI could be predicted using a single instrument such as the NMSS. CI in PD is, similar to other PD symptoms, heterogeneous and usually occurs concomitant with a variety of other NMS and associated burden of NMS (Goldman et al., 2018). Thus, a comprehensive method for quantifying PD manifestations such as CI in the context of other NMS is worthwhile, especially in prodromal stages (Martinez‐Martin, 2013). The NMSS encompasses practically and quantitatively the severity and frequency of NMS of patients with PD including items addressing functions related to cortex and limbic system. Also validated cutoffs for NMS burden have been published (Chaudhuri et al., 2007). We did not find significant differences in distribution of overall NMSB grading between the study groups but in specific NMS domains, which was confirmed in the logistic regression models. This is in line with the concept of several NMS dominant subtypes of PD, among which the limbic and cortical subtypes both encompass aspects of cognitive deficits (Sauerbier et al., 2016; Van Rooden et al., 2010; Zis et al., 2015). The cognitive aspect of nonmotor endophenotype in PD is also supported by prodromal studies, which suggest cognitive deficit in a subset (Weintraub et al., 2015) and also gut based cholinergic imaging studies (Knudsen et al., 2018).

Our results regarding significant higher baseline NMSS scores for hallucinations and perceptual problems (as reflected by the total scores for Doman 4 of the NMSS) are consistent with previous studies, which have shown that psychotic symptoms in PD, including delusions and hallucinations, are risk factors for the development of dementia and predictors of poor prognosis, mortality, and nursing home placement (Ffytche et al., 2017; Szatmari et al., 2017). The two groups in our study did not differ in disease duration and LED, so the difference in NMSS hallucinations/perceptual problems scores is unlikely to be the result of duration and dopaminergic medication dose. Indeed, studies from the prelevodopa era did mention hallucinations as part of disease manifestations (Fenelon et al., 2006). Besides, contrary to the results of other studies, in which sleep disorders were identified to be predictors of CI (Onofrj et al., 2002); we did not find any differences in overall PDSS scores between CA and CN groups at baseline. Moreover, we found that the patients in the CA group were significantly older, showed significantly higher SCOPA‐ME and ‐ADL scores and had a trend toward significantly more advanced HY stage compared with the patients in the CN group at baseline. These results are in line with previous studies, which provide clear evidence that age, motor impairment and measures of impairment in daily activities at baseline disease could predict the CI of patients (Zhu et al., 2014). Using Quade's rank analysis of covariance correction, we could show that the observed statistically significant higher NMSS domain 4 score in the patients of CA group was not due to age difference in the group, which suggest that hallucinations/perceptual problems might be initial manifestation of a subgroup of PD patients predisposed to CI and higher motor scores and age seem to be independent predictors, as also identified in our regression analyses.

Our analysis also revealed a trend toward significantly higher scores in the NMSS domain 5 (attention/memory) in the CA group compared to CN group at baseline. A 2‐step meta‐analysis comparing 30 neuropsychological tests of multiple cognitive domains showed that in nondemented PD patients memory, additionally to the more commonly reported domains of attention and executive function are impaired (Hoogland et al., 2018). This study is consistent with ours, as cognitive domains of memory and attention are addressed by the question in domain 5 of NMSS. In terms of other neuropsychiatric symptoms such as depression and anxiety measured by HADS at baseline, our analysis did not reveal any significant differences. These findings are not consistent with other studies indicating that depression and anxiety are predictors of CI in PD (De la Riva et al., 2014). Moreover, male sex has been proposed to be associated with CI as opposed to findings of our study, were no gender differences were found (Cammisuli et al., 2019). Our results suggest that the development of clinically relevant CI appears to be preceded by patient‐reported attention and memory problems before these can be objectified using formal cognitive assessment screening tools, such as the MMSE, but this phenomenon is not independent from age, gender, and the other baseline clinical characteristics of our cohorts.

A link between psychotic symptoms, attention/memory problem, and development of CI in nondemented PD patients has been reported (Knudsen et al., 2018). Cholinergic dysfunction appears to be a common pathophysiological mechanism, and cholinergic endophenotype of PD has been proposed (Aarsland et al., 2017; Bohnen & Albin, 2011; Müller & Bohnen, 2013). Neuropathological studies from PD patients with visual hallucinations showed atrophy in the pedunculopontine nucleus and nucleus basalis of Meyner (Janzen et al., 2012; Shin et al., 2012), which suggest the involvement of cholinergic system in the pathogenesis of hallucinations in PD. Moreover, in PD patients without a CI, such as the cohort of our study at baseline, lower cortical acetylcholinesterase positron emission tomography activity was associated with reduced cognitive performance scores for attention, memory, and executive functions (Aarsland et al., 2017). Our results may thus indicate that higher burden of hallucinations/ perceptual and attention/memory problems might be a marker for the “cholinergic endophenotype” of PD which has therapeutic connotations (Marras et al., 2020). In clinical practice, our findings suggest that, in patients with concomitant higher burden of perceptual and attention/memory problems, corresponding higher score in NMSS domain 4 and 5, the awareness of dementia development also in the next 3 years should be considered in relation to advanced planning and directive. Therefore, screening of PD patients with NMSS in addition to MMSE might be a useful method of predicting CI. This could have major potential clinical impact in relation to personalized medicine, enriching cohorts for neuroprotective studies, advanced directives as well as focused palliative care and caregiver support.

The retrospective design and a relatively short and variable follow‐up are the main limitations of this study, which should be addressed in future studies. For the diagnosis of idiopathic PD, the UK PD Brain Bank criteria were applied, because the start of data collection for our cohort dates back 2011, where the revised Movement disorder society (MDS) PD criteria (Postuma et al., 2015) were not available, and even now, some of the requisites for the 2015 MDS PD criteria such as objective testing of olfaction, cardiac metaiodobenzylguanidine (MIBG) scans are not routinely performed at diagnosis. In addition, we used only MMSE as an instrument to evaluate the CI in PD. This was because at the time of the setup of NILS in 2010, MMSE was recommended as the tool for cognitive assessment by the steering group of NILS. Despite its debatable accuracy and sensitivity, especially in mild cognitive deficits in PD patients, MMSE is still recommended as the primary screening instrument for PDD (Hoops et al., 2009) and used as a longitudinal test (Biundo et al., 2016). We used the threshold of 25 score of MMSE at endpoint follow‐up to dichotomize our cohorts and form the CA and CN groups. Scores under 25 are widely used to define the start of CI, relevant in daily life, and therefore fulfilled the main criterion of dementia, as recommended from the movement disorder society task for PDD (O'Bryant et al., 2008). At baseline, the MMSE score between the groups was also significant different, but not relevant in clinical practice as the difference was only 0.66 (mean) and MMSE score was above 28 as per inclusion criteria. The NMSS is a validated tool for assessing NMS in PD patients, reflecting a real‐world experience; however, NMSS contains only three items addressing cognitive domains, which are mainly assessed by history taking and are not an objective cognitive test. The strength of our study was that we studied a large number of patients (*n* = 541) and a diversity of variables regarding demographics and outcome of PD patients and we corrected for age and multiple comparisons.

To conclude, our results suggest that nonmotor profiling of PD patients by using the NMSS could be useful in aiding the prediction of CI development in PD patients over an average period of three years. Moreover, it can contribute to categorizing patients into a subgroup, where cholinergic systems might be pathophysiologically involved. High scores on the hallucinations/psychosis domain of the NMSS should alert the clinician to the likelihood that PD patients would develop CI over the coming years, preceding changes in more objective cognitive screening tools, such as the MMSE. In addition to sophisticated and detailed tools to predict CI, the NMSS system adds a pragmatic, quick win based strategy that can be widely applicable even in nonspecialized clinics.

## CONFLICT OF INTEREST

Dr. Oikonomou has been supported by the European Academy of Neurology Clinical Fellowship Programme 2019. Dr. van Wamelen reports grants and personal fees from Britannia Pharmaceuticals, personal fees from Invisio Pharmaceuticals, and personal fees from Abbvie. Dr. Weintraub has received research funding or support from Michael J. Fox Foundation for Parkinson's Research, Alzheimer's Therapeutic. Dr. Martinez‐Martin Research Initiative (ATRI), Alzheimer's Disease Cooperative Study (ADCS), the International Parkinson and Movement Disorder Society (IPMDS), and National Institute on Aging (NIA); honoraria for consultancy from Acadia, Aptinyx, Biogen, CHDI Foundation, Clintrex LLC, Eisai, Enterin, F. Hoffmann‐La Roche Ltd, Ferring, Janssen, Otsuka, Promentis, Sage, Signant Health, Sunovion, and Takeda; and license fee payments from the University of Pennsylvania for the QUIP and QUIP‐RS. Dr. Martinez‐Martin has received honoraria from National School of Public Health (ISCIII), Britannia, and Editorial Viguera for lecturing in courses, and from Bial, and Zambon for advice in clinical‐epidemiological studies. From the International Parkinson and Movement Disorder Society has received honoraria for management of the Program on Rating Scales, travel grant for attending the International Congress 2019, and financial support for development and validation of the MDS‐NMS. Dr Ffytche, Dr. Aarsland and Dr. Rodriguez‐Blazquez have nothing to disclose. Dr. Leta reports grants from Parkinson's UK, grants from Bial UK Ltd, other from Britannia pharmaceuticals, and other from Invisio Pharmaceuticals. Ms. Borley, Ms. Sportelli, Dr. Trivedi, Ms. Podlewska, Dr. Rukavina, and Dr. Lazcano‐Ocampo have nothing to disclose. Mrs. Rizos has received salary support from the National Institute of Health Research (NIHR) Clinical Research Network (CRN) South London and speaker honorarium from Britannia Pharmaceuticals Ltd. Dr. Ray Chaudhuri has received honoraria for advisory boards: AbbVie, Britannia Pharmaceuticals, UCB, Pfizer, Jazz Pharma, GKC, Bial, Cynapsus, Novartis, Lobsor, Stada, Medtronic, Zambon, Profile Pharma, Sunovion, Roche, Theravance Biopharma, Scion; honoraria for lectures from AbbVie, Britannia Pharmaceuticals, UCB, Mundipharma, Zambon, Novartis, Boeringer Ingelheim Neuroderm, Sunovion; grants (Investigator Initiated) from Britannia Pharmaceuticals, AbbVie, UCB, GKC, Bial, and academic grants from EU (Horizon 2020), IMI EU, Parkinson's UK, NIHR, PDNMG, Kirby Laing Foundation, NPF, MRC.

## AUTHOR CONTRIBUTION

Dr. Oikonomou conceptualized the work, analyzed and interpreted the date, drafted and critically reviewed the article, and approved the final version to be published. Dr. van Wamelen collected the date, analyzed and interpreted the date, drafted, and critically reviewed the article. Dr. Weintraub, Dr. Martinez‐Martin, Dr Ffytche, and Dr. Aarsland critically reviewed the article Dr. Rodriguez‐Blazquez analyzed the date and critically reviewed the article. Dr. Leta, Ms. Borley, Ms. Sportelli, Dr. Trivedi, Ms. Podlewska, Dr. Rukavina, Dr. Lazcano‐Ocampo, and Mrs. Rizos collected the date and critically reviewed the article. Dr. Ray Chaudhuri critically reviewed the article and approved the final version to be published.

### PEER REVIEW

The peer review history for this article is available at https://publons.com/publon/10.1002/brb3.2086.

## Data Availability

The data that support the findings of this study are available from the corresponding author, [Dr. Panteleimon Oikonomou], upon reasonable request.
